# When Communication Enables Catastrophe: Nuclear Risk and the Short Lifetimes of Technological Civilizations: A Bayesian Scenario Analysis of Civilizational Survival and the Fermi Paradox

**DOI:** 10.1111/risa.70339

**Published:** 2026-08-21

**Authors:** Vincenzo Alfano

**Affiliations:** ^1^ DISAE, University of Napoli “Parthenope” Naples Italy

**Keywords:** Bayesian modeling, civilizational collapse, command and control, existential risk, Fermi paradox, nuclear war, survival analysis, time‐varying hazards

## Abstract

The Fermi paradox may partly reflect technological self‐destruction acting as a Great Filter during post‐industrial development. Because the empirical record consists of a single civilization observed over an eighty‐year nuclear age, the exercise reported here is best understood as a structured, historically anchored scenario analysis rather than a data‐driven statistical estimate. We develop a time‐varying hazard model to estimate civilizational survival under nuclear risk, using Earth's documented close calls as an illustrative case. A Bayesian survival‐analysis framework with regime‐switching hazards captures temporal non‐stationarity between Cold War and post‐Cold War periods and propagates parameter uncertainty. Under high‐alert nuclear postures, estimated annual hazards range from roughly 0.1% to 2%, implying median survival times on the order of decades to millennia conditional on institutional configuration. These hazards plausibly exceed natural extinction risks but may decline substantially with improved governance and control systems. The results bound detectable civilizational longevity and offer conditional implications for existential‐risk prioritization and SETI expectations under deep uncertainty.

## 1 Introduction

The absence of observable extraterrestrial civilizations despite apparently favorable cosmic conditions, also known as the Fermi paradox (Webb 2015), has motivated numerous hypotheses spanning astrophysics, biology, and social science. Among these, Hanson's (1998) Great Filter hypothesis proposes that at least one evolutionary step from prebiotic chemistry to observable, space‐faring civilization is so difficult that virtually no species successfully navigates it. The filter's location remains profoundly uncertain: it may lie in our past (e.g., abiogenesis, eukaryogenesis, or multicellular evolution) or future (e.g., technological self‐destruction, artificial intelligence risks, or environmental collapse).

Determining filter location has critical implications for humanity's survival prospects. As Bostrom (2008) argues, “In the Great Silence there is Great Hope”: if we find no evidence of extraterrestrial life despite improving detection capabilities, this suggests we have successfully navigated the most difficult evolutionary steps. Conversely, discovering abundant microbial life or even complex biospheres would imply the Great Filter lies ahead, dramatically darkening humanity's long‐term outlook.

This article examines nuclear self‐destruction as one potential future filter, developing quantitative estimates of civilization survival time under specific institutional configurations. A central intuition motivating the analysis is that the technological threshold required for inter‐civilizational detectability may be closely coupled to the threshold required for civilization‐scale self‐destruction. The development of high‐energy physics, large‐scale industrial infrastructure, global communication networks, and advanced computation enables both sustained technosignature production and the capacity to generate existential hazards. If communicative and destructive capacities co‐emerge, civilizations may enter a fragile phase precisely when they become detectable. Our approach responds to methodological critiques in existential risk quantification (Ord et al. 2010; ÓhÉigeartaigh et al. 2020) by: (1) grounding estimates in formal stochastic processes (survival analysis, non‐homogeneous Poisson processes) rather than heuristic calculations; (2) employing regime‐switching models to capture temporal non‐stationarity; (3) using Bayesian scenario distribution structures to represent deep uncertainty and propagate it through credible intervals; and (4) framing conclusions conditionally with explicit scope limitations.

Existing quantitative studies of nuclear risk estimate the probability of war over specified time horizons, while discussions of the Fermi paradox generally rely on qualitative arguments about technological self‐destruction. To our knowledge, no previous framework has linked historically informed nuclear‐risk models to the detectable lifetime parameter of the Drake equation through an explicit survival‐analysis formulation. The purpose of this article is therefore not to estimate nuclear hazards but to provide a first step in this direction by offering a transparent probabilistic framework that connects these previously separate literatures under explicit scope conditions and deep uncertainty.

We emphasize at the outset what this analysis does not claim: it does not demonstrate that nuclear war is the Great Filter, nor that most civilizations follow Earth's developmental trajectory, nor that we can reliably extrapolate from a single civilization's 80‐year nuclear history to universal patterns. Rather, we pursue a more modest goal: developing a defensible quantitative framework for estimating survival times under well‐defined conditions, using Earth as an illustrative case to explore what such a filter might imply if certain political‐technical configurations prove common across technological civilizations.

Two further scope clarifications are stated here rather than deferred to later sections. First, the quantitative estimates reported below are direct transformations of assumed escalation probabilities rather than quantities learned from data: with only eight observed incidents, the posterior distributions presented are prior‐dominated, and the Markov Chain Monte Carlo (MCMC) machinery we employ serves chiefly to propagate parameter uncertainty forward rather than to extract information from the historical record. We return to this point explicitly when presenting results. Second, for Fermi paradox purposes, the failure event of interest is not nuclear exchange as such but what we call “detectability loss”: the transition from a technologically active civilization capable of producing observable technosignatures to a state in which such signals fall below plausible detection thresholds for external observers. Throughout the article we reserve the terms “nuclear hazard” and “civilizational hazard” for the hazard of nuclear exchange specifically, and we flag explicitly the conditional gap between that hazard and the hazard of detectability loss proper.

The article proceeds as follows. Section 2 provides a literature review covering the Great Filter hypothesis, existential risk quantification debates, nuclear war probability modeling, and civilizational collapse dynamics. Section 3 develops our methodological framework, including the survival analysis model, Bayesian scenario specification, and dataset construction criteria. Section 4 offers a summary of three pivotal incidents: the Cuban Missile Crisis (1962), the Petrov Incident (1983), and Able Archer 83 (1983), that justify the priors. Section 5 presents results including posterior hazard estimates, sensitivity analyses, and comparative risk assessment. Section 6 discusses scope conditions and conditional Fermi paradox implications. The last section concludes with implications for existential risk research and SETI.

## 2 Literature Review and Theoretical Framework

The Fermi paradox, commonly traced to Fermi's 1950 remark, highlights a tension between the apparent abundance of habitable worlds and the absence of observed extraterrestrial technological civilizations. The Drake equation formalizes this tension by decomposing the expected number of detectable civilizations into astrophysical, biological, and social parameters, among which the longevity of detectability, L, plays a decisive role (Drake 1961). The Great Filter hypothesis sharpens the inference: if detectable civilizations are rare, then at least one step from prebiotic chemistry to long‐lived technosignature production must be exceptionally improbable (Hanson 1998). Subsequent work refines this logic in two directions. First, anthropic and “hard‐step” arguments emphasize that observers necessarily appear only after passing prior bottlenecks, which complicates inference about where the most stringent filters lie (Carter 2008). Second, uncertainty‐forward treatments show that the “paradox” can be substantially weakened once wide parameter uncertainty is properly propagated, yet this does not remove the possibility that late‐stage, anthropogenic hazards sharply truncate L (Sandberg et al. 2018).

Within SETI research, the biosignature–technosignature distinction matters for locating filters. If life is common (biosignatures) but technology is absent (technosignatures), then the filter plausibly operates late, after life emerges but before durable technological activity persists (Wright et al. 2014; Haqq‐Misra et al. 2020). This makes the longevity component of the Drake equation not merely a residual term but a central empirical target, and it motivates explicit modeling of “detectable lifetime” rather than extinction alone.

This article's focus on nuclear self‐destruction connects to a broader methodological debate in existential‐risk research. Existential risks are commonly defined as threats that could annihilate intelligent life or permanently curtail its long‐term potential (Bostrom 2002). Estimating their probabilities is difficult because the relevant events are rare or unprecedented, mechanisms are complex, and the available evidence is fragmentary. Ord et al. (2010) distinguish broad families of approaches: frequency‐based inference (often infeasible for unprecedented hazards), expert elicitation (informative but bias‐prone), and structured modeling that links observable mechanisms to aggregate probabilities. More recent work emphasizes “deep uncertainty,” where the appropriate probability model is itself contested, and argues for uncertainty‐forward analysis rather than point estimates that can convey false precision (ÓhÉigeartaigh et al. 2020; Beard et al. 2020). Ord's (2020) synthesis offers influential headline estimates, but it also illustrates the central tension: these numbers are policy‐relevant, yet their evidentiary foundations mix sparse data, expert judgment, and modeling assumptions whose sensitivity is non‐trivial.

This debate has been carried forward substantially. Kent (2004) offers an early critical appraisal of global‐catastrophe risk assessments, cautioning against false precision in exactly the type of low‐probability, high‐stakes setting addressed here. More directly relevant to the present framework, Ćirković et al. (2010) show that observation‐selection effects systematically bias frequency‐based estimates of catastrophic risk: civilizations that experienced annihilating catastrophes are, by construction, absent from the observational record, so naively observed catastrophe rates understate true underlying hazards. This “anthropic shadow” result bears directly on our own catalogue of nuclear close calls, which by similar logic may undercount the true frequency and severity of near‐misses precisely because the worst realizations would have left no observers to record them (a point we return to in our discussion of survivorship bias in the conclusion). Ćirković’s later discussion of “small theories and large risks” reinforces the need to take low‐probability, high‐consequence mechanisms seriously even when theoretical confidence is limited (Ćirković 2012). In a way, the present article extends this line of work by translating such epistemic cautions into an explicit survival‐analysis framework for one candidate anthropogenic filter: nuclear self‐destruction.

Against this background, the nuclear‐war literature is especially instructive because it contains both (i) rich qualitative documentation of near‐miss mechanisms and (ii) a smaller but consequential quantitative tradition that attempts to translate those mechanisms into probabilities. Classical deterrence theory and crisis bargaining models clarify why nuclear crises generate escalation risk even under rationality and strategic interaction (Schelling 1960, 1966; Jervis 1979, 1989; Powell 1990; Fearon 1995). However, these approaches typically do not yield empirically grounded probability estimates. The empirical‐historical tradition, by contrast, documents the organizational and technical pathways to catastrophe, particularly failures in command‐and‐control, warning systems, and procedures under time pressure (Sagan 1993; Blair et al. 1993; Rhodes 2007). This literature repeatedly underscores the importance of compressed decision windows and the outsized role of individual judgment in averting escalation during specific episodes, a point that matters directly for any attempt to formalize hazard rates.

Quantitative estimates of inadvertent nuclear war risk span several methodological families, and their apparent disagreements can be reconciled once their target risk metrics are distinguished. First, frequency‐style calculations infer annualized risk from the historical incidence of nuclear‐armed years and documented close calls (Hellman 2008). Second, probabilistic risk assessment and fault‐tree approaches decompose escalation into interacting failure modes (e.g., warning errors, procedural breakdowns, and misinterpretation) and aggregate them into risk measures (Barrett et al. 2013; Baum et al. 2018). Third, Bayesian approaches combine structured priors with empirical or historical evidence, producing probability statements with credible intervals and sensitivity analysis. Here the contribution of Lundgren (2013) is central: it explicitly uses Bayesian updating to combine crisis‐level assessments and false‐alarm evidence to generate cumulative risk estimates over multi‐decade horizons, and it highlights how uncertainty spans orders of magnitude under alternative assumptions (Lundgren 2013). A related strand models specific pathways such as false‐alarm sequences under launch‐on‐warning postures; Wallace et al. (1986), using NORAD false‐alarm data, derive high crisis‐conditional probabilities for severe false‐alarm triggers during prolonged crises. Taken together, these studies motivate a key conceptual clarification: some estimates are “baseline annualized risks” averaged over peacetime and crisis periods, while others are “crisis‐conditional probabilities” that are not meant to be interpreted as year‐to‐year hazards (Baum et al. 2018; Wallace et al., 1986; Lundgren 2013). This distinction is essential for integrating heterogeneous quantitative findings without treating them as contradictory.

Two further points directly motivate the modeling choices in this article. First, several studies explicitly treat nuclear risk as time‐varying, driven by changes in arsenals, doctrines, warning systems, and geopolitical conditions rather than as a stationary process (Baum et al. 2018; Lundgren 2013). Second, methodological critiques caution that survival modeling in related domains is often undermined by distributional misspecification and untested assumptions (Piovani et al. 2021). These critiques reinforce the need for transparent scope conditions, explicit distributional choices, and sensitivity analysis when translating sparse incident catalogs into hazard estimates.

A striking gap, however, remains: most quantitative nuclear‐war studies estimate probabilities over specified time horizons or under particular crisis conditions, but they rarely translate those probabilities into explicit time‐to‐failure distributions for civilizational collapse or loss of detectability. A previous literature review identifies only limited attempts in this direction; Jiang et al. (2022) simulate survival time across multiple existential threats, including nuclear war, but do so using stylized distributional assumptions rather than survival functions grounded in close‐call processes and without a regime‐based hazard interpretation consistent with historical clustering. This gap is precisely where survival analysis can add value: a hazard framework yields a full survival distribution (and therefore a meaningful L in Drake‐equation terms) rather than only a probability statement at an arbitrarily chosen horizon.

Finally, civilizational‐collapse scholarship provides the bridge from “nuclear war probability” to “detectability loss.” Classical contributions emphasize general collapse mechanisms such as diminishing returns to complexity and fragility under growing systemic burdens (Tainter 1988), as well as environmental constraints and adaptive failures (Diamond 2005; McAnany and Yoffee 2010). Formal models such as HANDY couple population, resource dynamics, and inequalitydemonstrate pathways to systemic breakdown under specific structural conditions (Motesharrei et al. 2014). In the nuclear context, consequence modeling establishes that large‐scale nuclear conflict can produce global climatic and agricultural shocks through nuclear winter mechanisms (Robock et al. 2007; Toon et al. 2007; Toon et al. 2019; Robock et al. 2019). Work on post‐catastrophe resilience and alternative food systems further clarifies that “extinction” and “collapse” are not equivalent: substantial biological survival may coexist with long periods of technological regression (Denkenberger and Pearce 2014, 2015). For the Fermi paradox, this distinction is secondary to detectability, since prolonged technological loss is observationally similar to extinction from an external vantage point. This motivates modeling collapse (or detectability loss) as the relevant failure state for L while treating nuclear war as a mechanism that can trigger that transition under specific institutional configurations.

Recent work on island refuges under severe nuclear‐winter conditions (Kallenborn and Willis 2025) shows that catastrophic risk analysis need not stop at the initiating event; it can also examine pathways for resilience, partial survival, and technological recovery. This distinction is central here: the relevant SETI quantity is not human extinction alone but the persistence or loss of detectable technological activity.

In sum, existing work provides the conceptual components needed to formalize nuclear self‐destruction as a candidate late‐stage filter: the Drake‐equation emphasis on detectable lifetime (Drake 1961; Wright et al. 2014), the Great Filter framing (Hanson 1998; Carter 2008), existential‐risk methodology under deep uncertainty (Ord et al. 2010; ÓhÉigeartaigh et al. 2020; Beard et al. 2020), quantitative nuclear‐war risk estimation with explicitly time‐varying and mechanism‐based perspectives (Lundgren 2013; Wallace et al. 1986; Barrett et al. 2013; Baum et al. 2018), and collapse consequence pathways relevant to detectability (Robock et al. 2019; Denkenberger and Pearce 2014, 2015). The remaining methodological step, and one of this article's contributions, is to combine these elements into a transparent survival‐analysis framework that (i) represents regime non‐stationarity, (ii) propagates uncertainty through Bayesian priors, and (iii) yields a survival distribution interpretable as a bound on detectable civilizational longevity under specified scope conditions.

## 3 Methodology: Survival Analysis With Time‐Varying Hazards

We model civilization survival as a continuous‐time stochastic process characterized by a hazard function λ(t), representing the instantaneous risk of catastrophic failure at time t conditional on having survived until t. Following standard survival analysis theory (Cox 1972; Kalbfleisch and Prentice 2002), we define the following:

$$\mathrm{Hazard}\mathrm{function}:\lambda \left(t\right)=\lim_{\Delta t\to 0}P\left(t\leq T<t+\Delta t|T\geq t\right)/\Delta t$$

where *T* is the random variable representing time until catastrophe. The hazard function captures the instantaneous failure rate given survival up to time t.

$$\mathrm{Survival}\mathrm{function}:S\left(t\right)=P\left(T>t\right)=\exp\left(-\int_{0}^{t}\lambda \left(\tau \right)d\tau \right)$$

This gives the probability of surviving beyond time t. The integral in the exponent is the cumulative hazard.

$$\mathrm{Cumulative}\mathrm{hazard}:\Lambda \left(t\right)=\int_{0}^{t}\lambda \left(\tau \right)d\tau$$

so that S(t) = exp(‐Λ(t)). The cumulative hazard represents total accumulated risk over the interval [0,t].

$$\mathrm{Probability}\mathrm{density}:f\left(t\right)=\lambda \left(t\right)S\left(t\right)=\lambda \left(t\right)\exp\left(-\Lambda \left(t\right)\right)$$

representing the probability density of catastrophe occurring at exactly time t.

For constant hazard λ, the survival function simplifies to S(t) = exp(‐λt), yielding an exponential distribution with mean survival time E[T] = 1/λ and median t_median_ = ln(2)/λ ≈ 0.693/λ. However, our empirical observations violate constant‐hazard assumptions, motivating time‐varying specifications.

The median survival time is defined by S(t_median_) = 0.5. For constant hazard λ:

$$\exp\left(-\lambda t_{\mathrm{median}}\right)=0.5$$

$$-\lambda t_{\mathrm{median}}=\ln\left(0.5\right)$$

$$t_{\mathrm{median}}=-\ln\left(0.5\right)/\lambda =\ln\left(2\right)/\lambda$$

Since ln(2) ≈ 0.693, we have t_median_ ≈ 0.693/λ. This relationship allows us to convert between hazard rates and survival times.

We model nuclear close calls as realizations of a non‐homogeneous Poisson process with intensity function μ(t), representing the rate at which crisis incidents occur. Each incident carries the conditional escalation probability *p_esc_*, that is, the probability that, given an incident occurs, it escalates to a full nuclear exchange.

The civilizational hazard rate becomes:

$$\lambda \left(t\right)=\mu \left(t\right)\times p_{\mathrm{esc}}$$

This decomposition separates observable quantities (incident frequency μ(t), which we can estimate from historical data) from fundamentally unobservable counterfactuals (escalation probability *p_esc_*, about which we can only make informed assumptions). The current model abstracts from mechanism‐specific escalation probabilities and instead treats p*_esc_* as a regime‐level mixture parameter summarizing heterogeneous escalation pathways.

If we observe N incidents over time period [0,T] and assume incidents follow a Poisson process with constant intensity μ, the likelihood function is:

$$L\left(\mu |N,T\right)={\left(\mu T\right)}^{N}\exp\left(-\mu T\right)/N!$$

The maximum likelihood estimate is μ̂ = N/T. However, this assumes perfect observation. If we only observe fraction α of true incidents (due to classification, incomplete records, or definitional exclusions), the true intensity is μ_true_ = μ̂/α = N/(αT).

Combining these elements:

$$\lambda =\left(N/\alpha T\right)\times p_{\mathrm{esc}}$$

This provides the connection between observable data (N, T) and target quantity (λ), mediated by unobservable parameters (α, p*_esc_*).

Earth's nuclear history exhibits stark temporal clustering. Seven of the eight cataloged incidents occurred during the Cold War period 1950–1991 (Korean War threats, Suez Crisis, Cuban Missile Crisis, Yom Kippur War, NORAD false alarm, Petrov incident, and Able Archer 83). One incident, the Norwegian rocket incident of January 1995, falls strictly after the 1991 regime boundary and therefore belongs to the post‐Cold War regime under a literal application of the stated cutoff, giving N_CW  =  7 and N_post  =  1. No qualifying incidents occurred between 1996 and 2020. This temporal pattern suggests regime‐dependent hazards rather than temporal homogeneity, and we adopt N_CW  =  7, N_post  =  1 throughout.

We model this using piecewise‐constant hazards:

$$\lambda \left(t\right)=\lambda_{\mathrm{CW}}\times 1_{\left[t\in \mathrm{Cold}\mathrm{War}\right]}+\lambda_{\mathrm{post}}\times 1_{\left[t\in \mathrm{post}-\mathrm{CW}\right]}$$

where 𝟙 represents indicator functions. For regime j with duration T_j_ and N_j_ observed incidents:

$$\lambda_{j}=\left(N_{j}/\alpha_{j}T_{j}\right)\times p_{\mathrm{esc},j}$$

The cumulative hazard over both regimes (defining t_CW_ as the Cold War end date):

$$\Lambda \left(t\right)=\lambda_{\mathrm{CW}}\times T_{\mathrm{CW}}+\lambda_{\mathrm{post}}\times \left(t-T_{\mathrm{CW}}\right)\mathrm{for}t>T_{\mathrm{CW}}$$

And survival probability:

$$S\left(t\right)=\exp\left(-\lambda_{\mathrm{CW}}\times T_{\mathrm{CW}}-\lambda_{\mathrm{post}}\times \left(t-T_{\mathrm{CW}}\right)\right)$$

This allows distinct hazard levels in different eras while maintaining proper probability structure.

Given fundamental uncertainty about unobservable parameters (p*_esc_*, α), we use Bayesian uncertainty propagation to generate scenario distributions for the implied hazard rates and survival times from the specified prior distributions for α, p*_esc_*, and *r*.

We use α ∼ Beta(a, b). The Beta distribution is natural for probabilities, with shape parameters determining the prior mean a/(a+b) and concentration. We specify three scenarios:

- Optimistic: Beta(3, 2), mode = 0.67, mean = 0.6, indicating belief we observe most incidents
- Moderate: Beta(2, 2), relatively flat on [0,1], mean = 0.5
- Pessimistic: Beta(2, 4), mode = 0.25, mean = 0.33, substantial underreporting

Given p*_esc_* ∈ (0,1) with positive skew, we use log‐normal: log(p*_esc_*) ∼ Normal(µ_log_, σ_log_). This implies:

$$E\left[p_{\mathrm{esc}}\right]=\exp\left(\mu_{\log}+\sigma^{\log2}/2\right)$$

$$\mathrm{median}\left[p_{\mathrm{esc}}\right]=\exp\left(\mu_{\log}\right)$$

- Optimistic: µ_log_ = ‐5.5, σ_log_ = 1.0 → median = 0.4%, 90% CI [0.06%, 2.7%]
- Moderate: µ_log_ = ‐4.5, σ_log_ = 1.5 → median = 1.1%, 90% CI [0.1%, 12%]
- Pessimistic: µ_log_ = ‐3.5, σ_log_ = 1.2 → median = 3%, 90% CI [0.5%, 18%]

A log‐normal distribution has support (0,∞) and therefore formally assigns positive probability to *p_esc_* > 1, which is not meaningful for a probability. Given the parameterizations above, this mass is negligible (all reported 90% upper bounds remain below 1), so the practical impact on posterior results is minimal; nonetheless, a logit‐normal or truncated log‐normal specification would be the mathematically proper choice and is a candidate refinement for future iterations of this model.

We use r ∼ Beta(2, 5), mode = 0.2, and mean = 0.29, encoding the belief that post‐Cold War risks decreased substantially but did not vanish. The 90% credible interval [0.05, 0.60] allows for risk reductions ranging from 40% to 95%.

We generate Bayesian scenario distributions for λ_CW_ and λ_post_ by jointly sampling the uncertain parameters α, p*_esc_*, and r using MCMC and propagating each draw through the hazard equations defined above. Throughout the analysis, we assume that catalogue completeness (α) and the conditional escalation probability (p*_esc_*) are common across the Cold War and post‐Cold War regimes, while the post‐Cold War hazard is obtained by scaling the Cold War hazard according to

$$>\lambda_{\mathrm{post}}=r,\lambda_{\mathrm{CW}\cdot }>$$

Consequently, the post‐1991 catalog is not used to estimate a separate hazard process but rather to motivate the choice of a reduced‐hazard scenario represented by r. From the sampled hazard distributions we compute the following derived quantities:

- Median survival time: t^median(i)^ = ln(2) / λ^CW(i)^
- 90% credible intervals: 5th and 95th percentiles of {t^median(i)^}
- Probability that λ_post_ < λ_CW_: fraction of samples where regime improvement occurred

A central challenge in Bayesian analysis under deep uncertainty is the specification of priors for parameters that are not directly observable. In the present context, escalation probability (p*_esc_*), catalogue completeness (α), and regime hazard reduction (r) cannot be inferred from repeated empirical observations. We therefore ground prior choices in three complementary sources: historical escalation assessments during documented crises, quantitative estimates from nuclear‐risk modeling literature, and conservative uncertainty‐forward principles commonly adopted in existential‐risk analysis.

Historical evidence provides partial constraints on plausible escalation magnitudes. During the Cuban Missile Crisis, contemporaneous assessments by senior decision‐makers placed the probability of nuclear war between roughly one‐third and one‐half, reflecting extreme political confrontation combined with incomplete information and compressed timelines. By contrast, technical false‐alarm incidents such as the 1979 NORAD malfunction or the 1983 Petrov episode are generally interpreted as substantially lower‐probability escalation pathways, since multiple verification layers and decision delays remained available. These contrasts support a wide but bounded prior range for p*_esc_* spanning approximately 10^−^^3^ to 10^−^^1^, consistent with both crisis‐level and technical‐failure scenarios.

Independent modeling studies reach comparable orders of magnitude. Probabilistic risk assessments of inadvertent nuclear war during high‐alert postures commonly produce annual war probabilities in the range of 10^−^^3^ to 10^−^^2^, depending on assumptions about false alarms, command‐and‐control failures, and crisis instability. While these estimates remain deeply uncertain, they provide external validation that the prior ranges adopted here are not arbitrarily chosen but lie within the spectrum considered plausible in the specialist literature.

Prior beliefs about catalog completeness (α) derive from structural features of nuclear secrecy rather than numerical evidence. Classification practices, incomplete archival access in several nuclear‐armed states, and definitional ambiguity regarding “close calls” jointly imply systematic under‐observation. A symmetric Beta prior centered near 0.5, therefore, encodes agnosticism while allowing substantial probability mass across severe undercounting and near‐complete observation. Similarly, the prior on‐regime hazard reduction (r) reflects qualitative historical judgment that post–Cold War institutional changes likely reduced but did not eliminate nuclear risk.

Taken together, these considerations motivate weakly informative but historically anchored priors: wide enough to represent genuine epistemic uncertainty, yet constrained by empirical plausibility and existing quantitative scholarship. Sensitivity analysis reported later on confirms that the article's central qualitative conclusion, that is, that plausible nuclear hazards exceed natural extinction risks by multiple orders of magnitude, remains robust across substantial prior variation.

We compiled a structured catalogue using criteria designed to balance inclusiveness with evidentiary rigor, from 1945 to 2020, covering the entire nuclear age from the Trinity test through the present, about incidents involving declared nuclear‐armed states (United States, USSR/Russia, United Kingdom, France, and China) or undeclared but confirmed nuclear powers (Israel, India, Pakistan, and North Korea). Incidents included have to meet at least one criterion: (a) nuclear forces placed on heightened alert (DEFCON 3 or higher, equivalent Soviet/Russian readiness), (b) false attack warnings generated by early warning systems, or (c) explicit nuclear threats issued by state leaders during active crises. Primary sources (declassified government documents, official military records, and verified transcripts) or secondary scholarly sources with clear documentation chains citing primary materials are used. We exclude incidents based solely on single‐source journalism, memoirs without documentary support, or unverified claims. Contemporary actors or subsequent scholarly analysis must indicate non‐negligible escalation probability. These conservative criteria mean our N er  8 total incidents (N_CW  =  7, N_post  =  1) likely underestimate true close‐call frequency, implying α < 1.0.

The historical record reveals several structural patterns relevant for hazard modeling. First, nuclear close calls exhibit pronounced temporal clustering, with all documented incidents occurring between 1950 and 1995 and none meeting inclusion criteria in the subsequent 25 years, a regime discontinuity that motivates the use of time‐varying hazard rates. Second, escalation mechanisms are heterogeneous, spanning deliberate geopolitical brinkmanship, technical system failures, and misinterpretation of military exercises, indicating that vulnerability does not arise from a single dominant pathway but from multiple interacting failure modes. Third, individual human agency repeatedly proves decisive, as several incidents were averted only through discretionary decisions that departed from established protocol, implying that civilizational survival may partially depend on contingent personal judgment rather than solely institutional design. Finally, escalation risk is intensified by severe compression of decision timelines: technical false alarms reduce response windows to minutes, while political crises allow only hours or days, both dramatically shorter than normal strategic deliberation. Together, these features support modeling nuclear risk as a regime‐dependent, mechanism‐diverse hazard shaped by institutional structure and temporal urgency rather than as a stationary or purely random process.

## 4 Mechanisms of Nuclear Escalation: Evidence From Close‐Call Case Studies

This section distills key escalation mechanisms from historically documented nuclear close calls, focusing on how institutional configurations, decision windows, and information failures translate into non‐negligible escalation probabilities. Rather than providing detailed historical narratives, we extract features relevant for hazard modeling and prior specification. Case descriptions are reported in Appendix A.

Across cases, three recurring escalation channels emerge: (i) political crises with compressed decision timelines, (ii) technical false alarms interacting with launch‐on‐warning postures, and (iii) misinterpretation of adversary intent during military exercises. These channels differ in surface characteristics but share a common structure: extreme asymmetry between the speed of potential catastrophe and the slower pace of error correction.

The Cuban Missile Crisis (1962) represents the clearest instance of political escalation risk. Senior decision‐makers on both sides faced incomplete information, ambiguous signals, and intense time pressure, with nuclear forces placed on high alert. Contemporary assessments by U.S. leadership placed the probability of nuclear war at substantial levels, indicating that escalation was not perceived as a remote tail event but as a central policy concern. This case anchors the upper range of plausible escalation probabilities (p*_esc_*) during acute geopolitical crises.

Similar dynamics, though at lower intensity, are observable during the Korean War and the Yom Kippur War, where explicit nuclear threats or force posturing occurred under conditions of regional conflict and alliance uncertainty. These cases suggest that escalation probability is state‐dependent, rising sharply during periods of geopolitical confrontation.

A second escalation pathway arises from technical malfunction within early warning and command‐and‐control systems. The 1979 NORAD training tape incident and the 1983 Soviet satellite false alarm demonstrate how erroneous signals of incoming attack can compress decision windows to minutes. In both cases, nuclear retaliation was actively prepared before human judgment intervened.

These incidents highlight that escalation probability does not depend solely on adversarial intent. Instead, system reliability, alert posture, and verification protocols play decisive roles. While p*_esc_* in such cases is plausibly lower than in full political crises, the high frequency of false alarms during early technological phases implies that cumulative risk remains substantial.

The Able Archer 83 exercise and the Norwegian rocket incident illustrate escalation driven by misinterpretation rather than malfunction. In these cases, routine or scientific activities were interpreted as potential first‐strike preparations, triggering heightened alert responses. Escalation risk arose not from explicit hostile action but from ambiguity combined with doctrinal expectations under launch‐on‐warning systems. These cases reinforce the importance of institutional context: identical signals can be interpreted benignly or catastrophically depending on prevailing threat perceptions and strategic doctrines.

Taken together, these cases justify three modeling assumptions adopted in this paper. First, escalation probabilities are heterogeneous across incident types but plausibly span several orders of magnitude, motivating wide but bounded priors on p*_esc_*. Second, incident frequency and escalation risk are jointly shaped by institutional design, particularly alert posture and decision centralization. Third, temporal clustering of incidents during the Cold War reflects regime‐level vulnerability rather than random fluctuation, supporting the use of time‐varying hazard rates.

The case evidence, therefore, functions not as an anecdotal illustration,∖ but as structural grounding for the probabilistic framework employed below. Narratives and documentary sources are provided in Appendix A to preserve transparency while maintaining analytical focus in the main text.

## 5 Results: Benchmark Hazards and Bayesian Posterior Survival Projections

The quantitative results presented here are conditional on the modeling assumptions and the prior structures detailed above. Because nuclear close calls cannot be repeatedly observed and humanity provides the only example of a technological civilization, the framework does not produce frequentist long‐run probabilities. Instead, it combines historically anchored priors with a stochastic hazard structure to explore plausible survival implications under specified institutional configurations. Results therefore bound detectable longevity under explicit assumptions rather than forecast precise extinction timelines.

To clarify interpretation, we present two complementary summaries: (i) deterministic plug‐in hazard benchmarks based on central parameter values, and (ii) full Bayesian posterior results integrating parameter uncertainty.

### 5.1 Deterministic Hazard Benchmarks (Central‐Parameter Estimates)

As a transparent baseline, we first evaluate the hazard formula.

$$\lambda =\frac{N}{\alpha T}\;\cdot p_{esc}$$

Because the historical catalogue contains only eight qualifying incidents, we do not attempt to estimate latent incident intensities through a separate likelihood model. Instead, we specify prior distributions for the uncertain parameters α (prior mean), p*_esc_* (prior median), and r; draw jointly from these distributions using MCMC, and propagate the resulting uncertainty through the deterministic hazard transformation above. The resulting distributions therefore represent Bayesian scenario distributions under the stated assumptions rather than likelihood‐dominated posterior learning from the incident record.

For the Cold War period (1950–1991, T = 41 years, N_CW = 7 incidents), this yields:

*Optimistic scenario* α ≈ 0.6, median p*_esc_* ≈ 0.4%

λ ≈ 0.00114 per year

Median survival ≈ 609 years

*Moderate scenario* α ≈ 0.5, median p*_esc_* ≈ 1.1%

λ ≈ 0.00376 per year

Median survival ≈ 184 years

Pessimistic scenario α ≈ 0.33, median p_esc_ ≈ 3%

λ ≈ 0.0155 per year

Median survival ≈ 45 years

These values provide intuitive benchmarks under central assumptions. Even in optimistic configurations, implied detectable lifetimes are measured in centuries rather than geological timescales.

However, these plug‐in estimates ignore parameter uncertainty and nonlinear propagation effects. We therefore turn to the full scenario distribution.

### 5.2 Bayesian Posterior Hazard Distributions

Using (MCMC) sampling of the Bayesian scenario model described, we obtain scenario distributions for λCW and λpost by propagating uncertainty in:

- Escalation probability (p*_esc_*),
- Catalog completeness (α),
- Regime reduction parameter (r).

Across scenarios, median hazard estimates from the Bayesian scenario distributions lie in the range 10^−^^3^–10^−^^2^ per year, with wide but finite 90% credible intervals spanning roughly an order of magnitude within each scenario.

Two features are noteworthy. First, posterior medians are generally close to the deterministic benchmarks but not identical. Because λ depends multiplicatively on uncertain parameters, the scenario distribution is right‐skewed, and Jensen‐type effects shift the median relative to plug‐in values.

Second, credible intervals are broad. Survival distributions derived from λ are strongly right‐skewed: while long lifetimes remain possible under optimistic parameter combinations, non‐trivial probability mass lies on relatively short horizons (decades to low centuries).

Transforming sampled hazard draws into survival functions S(t) = exp(−λt) yields median detectable lifetimes ranging from decades (pessimistic) to several centuries or longer (optimistic), with upper credible bounds extending into millennial scales. This constant‐hazard transformation is applied within a single regime (Cold War or post‐Cold War) and is exact there; for horizons spanning both regimes, the cumulative‐hazard survival function S(t) = exp[−λ_CW × T_CW − λ_post × (t−T_CW)] introduced above, not the single‐regime exp(−λt) form, applies.

**FIGURE 1:**
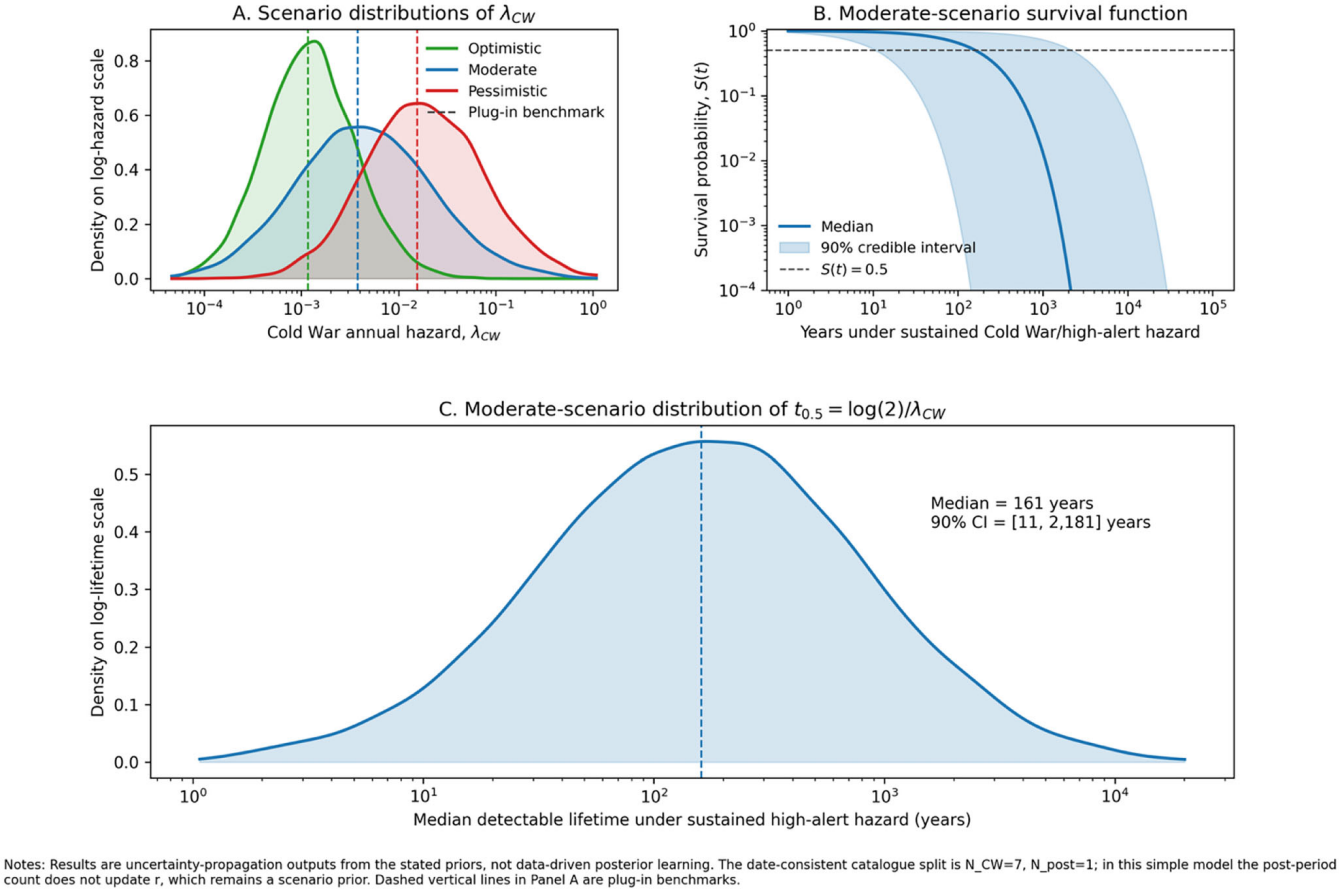
Bayesian scenario distributions and survival projections under alternative prior scenarios. *Note*: Panel A shows Bayesian scenario distribution of Cold War annual hazard rates (λCW) under optimistic, moderate, and pessimistic prior specifications, plotted on a logarithmic scale. Dashed vertical lines indicate deterministic plug‐in benchmarks based on prior means and medians. Scenario distributions are right‐skewed and span approximately one order of magnitude within scenarios. Panel B displays the implied survival function under the moderate scenario, with median survival curve and 90% credible band derived from posterior hazard draws. The widening band illustrates the cumulative effect of parameter uncertainty over time. These distributions summarize uncertainty propagation from the stated prior assumptions rather than likelihood‐dominated posterior learning.

Figure 1 visualizes the scenario distributions of Cold War hazard rates across the three prior scenarios, together with the corresponding deterministic plug‐in benchmarks. The log‐scale representation highlights the right‐skewed structure of the posterior and the order‐of‐magnitude separation between optimistic, moderate, and pessimistic configurations. While deterministic benchmarks lie near the central posterior mass, uncertainty propagation generates substantial dispersion, particularly in the upper tail. Panel B translates these hazard distributions into survival functions under the moderate scenario, illustrating how parameter uncertainty maps into widening credible bands over time. The figure therefore makes explicit that conclusions are driven by broad distributional structure rather than isolated point estimates. Notably, even the lower‐density regions of the optimistic posterior exceed typical natural extinction hazard magnitudes by multiple orders of magnitude, reinforcing the robustness of the comparative risk conclusion.

The post‐1991 period includes one qualifying incident over 29 years (the Norwegian rocket incident, January 1995; N_post  =  1). A single post‐1991 incident is broadly consistent with a substantially reduced but non‐zero hazard. Under the regime‐switching model with r ∼ Beta(2,5), the scenario distributions continue to indicate substantial hazard reduction under the assumed prior on r.

Posterior medians for λpost are typically lower by a factor of approximately 3–10 relative to Cold War values, depending on scenario. Survival medians shift accordingly toward multi‐century or longer horizons, though credible intervals remain wide and partially overlapping with earlier risk levels. The single post‐1991 event over 29 years is statistically compatible with moderate hazard reductions; it does not provide strong evidence for either elimination or persistence of Cold War‐level risks.

Natural catastrophic risks (large asteroid impacts, supervolcanic eruptions) are typically estimated at annual hazard rates on the order of 10^−^^6^–10^−^^5^. Across both deterministic benchmarks and scenario distributions, Cold War nuclear hazard estimates exceed these magnitudes by several orders of magnitude under moderate and pessimistic scenarios.

Even under optimistic assumptions, the lower credible bounds for nuclear risk approach or exceed combined natural hazards. While such comparisons require caution, given methodological differences between geological frequency inference and escalation modeling, the order‐of‐magnitude separation persists across reasonable parameter ranges.

Given sparse data (7 Cold War incidents, 1 post‐1991), posterior results remain prior‐influenced. With N_CW  =  7 and N_post  =  1, the posterior variance is dominated by prior structure rather than likelihood concentration. The framework therefore serves as a structured scenario analysis rather than data‐driven estimation. However, sensitivity analysis indicates:

- Increasing median p*_esc_* by an order of magnitude shifts survival medians proportionally but does not eliminate century‐scale vulnerability.
- Varying α between high completeness (≈0.9) and substantial undercounting (≈0.3) alters hazards by factors of 2–3.
- Broad, weakly informative priors expand credible intervals substantially but do not reverse the qualitative conclusion that plausible nuclear hazards exceed natural background risks.

Thus, while numerical precision is limited, the qualitative ordering of risk magnitudes remains robust across substantial prior variation.

MCMC diagnostics were obtained using the No‐U‐Turn Sampler (NUTS) implemented in PyMC 6.0, and are presented in Table 1. For each scenario, four independent chains were run with 2000 warm‐up iterations and 3500 retained draws per chain, using a target acceptance probability of 0.95. All twenty‐one monitored quantities achieved split ∖hat{R} values of 1.000. Bulk effective sample sizes ranged from 5967 to 12,613, while tail effective sample sizes ranged from 3299 to 8968. No divergent transitions occurred in any scenario. Trace plots and rank histograms for the three sampled parameters (α, p*_esc_*, and *r*) are provided in Supplementary Figures S1–S6 and show no evidence of chain‐specific behavior or poor mixing. Taken together, these diagnostics indicate computationally stable exploration of the specified Bayesian scenario distributions.

**TABLE 1:** NUTS convergence diagnostics.

Scenario	Quantity	R‐hat	ESS (bulk)	ESS (tail)	Divergences
Optimistic	α	1.000	12,379	7959	0
Optimistic	p*_esc_*	1.000	11,236	6929	0
Optimistic	r	1.000	11,330	7461	0
Optimistic	λ_CW_	1.000	11,345	8136	0
Optimistic	λ_post_	1.000	10,554	7709	0
Optimistic	tmed_CW_	1.000	11,345	8136	0
Optimistic	tmed_post_	1.000	10,554	7709	0
Moderate	α	1.000	12,535	8968	0
Moderate	p*_esc_*	1.000	7428	4171	0
Moderate	r	1.000	12,613	7577	0
Moderate	λ_CW_	1.000	8074	4465	0
Moderate	λ_post_	1.000	8486	5675	0
Moderate	tmed_CW_	1.000	8074	4465	0
Moderate	tmed_post_	1.000	8486	5675	0
Pessimistic	α	1.000	10,229	6955	0
Pessimistic	p*_esc_*	1.000	5967	3299	0
Pessimistic	r	1.000	10,738	7939	0
Pessimistic	λ_CW_	1.000	6788	4332	0
Pessimistic	λ_post_	1.000	6906	4940	0
Pessimistic	tmed_CW_	1.000	6788	4332	0
Pessimistic	tmed_post_	1.000	6906	4940	0

*Note*: Diagnostics from the No‐U‐Turn Sampler (NUTS) implemented in PyMC 6.0. Four independent chains were run for each prior scenario, with 2000 warm‐up iterations and 3500 retained draws per chain (target acceptance probability = 0.95). R‐hat denotes the split potential scale reduction statistic; ESS denotes the effective sample size. No divergent transitions occurred in any run.

Because the model propagates uncertainty from explicitly specified prior distributions rather than attempting to estimate poorly identified latent quantities from a sparse incident catalog, the resulting distributions should be interpreted as scenario distributions conditional on the stated assumptions. The excellent NUTS diagnostics therefore demonstrate reliable exploration of the specified scenario distributions, rather than strong empirical identification of the underlying parameters.

The deterministic plug‐in benchmarks provide transparent central‐case illustrations. The Bayesian posterior generalizes these by incorporating uncertainty and revealing skewed survival distributions. Together, they indicate that under high‐alert nuclear configurations, detectable civilizational lifetimes plausibly lie in the range of decades to centuries, rather than millennia or longer.

These results remain conditional on Earth‐like institutional and technological structures. They do not imply that nuclear war is the Great Filter, nor that most civilizations follow Earth's trajectory. They demonstrate instead that if certain configurations are common, time‐varying nuclear hazard alone can truncate detectable longevity sufficiently to materially affect the Drake equation's L parameter.

## 6 Scope Conditions and Conditional Fermi Paradox Implications

Our hazard estimates apply specifically to civilizations exhibiting Earth's Cold War configuration. It includes some characteristics. First, geopolitical bipolarity or multipolarity, for example, competing power centers with existential rivalry. Unified planetary governance might avoid nuclear deterrence dynamics entirely, and hence to extend to each civilization geopolitical bipolarity might be an unsustainable assumption. Second, launch‐on‐warning postures. Hair‐trigger alert systems compressing decision windows to minutes. Alternative doctrines (assured second‐strike without launch‐on‐warning and no‐first‐use) might substantially reduce accident risks. Third, early‐generation early warning, characterized by technical unreliability during initial nuclear epoch. More mature sensor networks with multiple redundant channels could reduce false alarm frequency. Fourth, centralized command authority: single individuals or small committees with launch authority. Distributed veto structures might increase robustness. Finally, incomplete communication channels. Limited crisis communication mechanisms, while robust verification protocols might reduce misperception‐driven escalation.

Crucially, these conditions are not necessarily universal. Alternative developmental pathways might avoid some or all of these vulnerabilities, reducing the external validity of the analysis.

A further, more fundamental scope condition concerns the physical character of the destructive technology itself rather than the institutional configuration surrounding it. The escalation‐probability priors developed are anchored in the historical record of nuclear fission and fusion weapons, the only existential‐capable weapon class for which Earth provides direct evidence. Nothing in the analysis establishes, or could establish from a sample of one, that the physics available to another technological civilization would yield comparably destructive technologies of a similar kind. An alternative physical regime might permit existential‐scale weapons with entirely different escalation dynamics, decision‐time compression, or command‐and‐control vulnerabilities than those documented, or it might permit no single‐event existential weapon at all, in which case the nuclear window mechanism we describe would simply not apply. Generalizing our survival estimates beyond civilizations whose destructive capacities resemble nuclear weapons not merely in degree but in kind therefore requires an additional, untested assumption about the convergence of weapons physics across different planetary histories, which we flag here as a limitation rather than leave implicit.

Existential‐risk discussions often distinguish between human extinction (complete biological annihilation) and civilizational collapse (loss of technological, institutional, and knowledge systems sufficient to sustain advanced society). For the purposes of the Fermi paradox and technosignature detectability, this distinction requires further refinement. What matters observationally is not biological survival per se, but the persistence of detectable technological activity over astronomical timescales.

We therefore introduce the concept of detectability loss, defined as the transition from a technologically active civilization capable of producing observable technosignatures (e.g., radio emissions, atmospheric industrial signatures, large‐scale energy use) to a state in which such signals fall below plausible detection thresholds for external observers. Detectability loss may occur through complete extinction, prolonged civilizational collapse, or durable technological regression lasting longer than the characteristic lifetime of technosignatures.

Formally, let

- T_e_ denote time to biological extinction,
- T𝚌 denote time to civilizational collapse, and
- T_d denote time to detectability loss.

These satisfy the weak ordering:

$$T\_d\leq T_{C}\leq T_{e}$$

because detectability can cease even if small human populations survive, and collapse can precede extinction by centuries or millennia. For SETI‐relevant longevity, the operative quantity in the Drake equation is therefore T_d, not extinction time. Crucially, the technologies that render civilizations detectable (high‐power radio transmission, large‐scale energy generation, and advanced physics and engineering) may be developmentally adjacent to those enabling existential self‐destruction. This adjacency implies that the onset of detectability may coincide with entry into a high‐hazard regime, creating a narrow temporal overlap window between independent civilizations.

Under nuclear‐winter scenarios modeled in the geophysical literature, large‐scale agricultural failure and institutional breakdown could plausibly eliminate global technological activity for extended periods even while permitting partial human survival. In such cases, civilizational hazard rates estimated in this article map naturally onto detectability hazard, reinforcing the relevance of nuclear risk to Great Filter hypotheses without requiring assumptions about total extinction.

This clarification also bounds interpretation. A civilization might recover technologically after collapse, implying that detectability loss is not necessarily permanent. However, if recovery times exceed typical intervals between independent catastrophic risks, repeated collapse events could still limit long‐term observable longevity. The framework adopted here therefore treats nuclear hazard as governing the effective detectable lifetime of technological civilizations rather than their absolute biological duration.

We make this gap explicit rather than leave it implicit. Let p_DL|war denote the conditional probability that a full‐scale nuclear exchange produces detectability loss as defined above, as opposed to a less severe outcome from which detectable technological activity recovers on a timescale short relative to typical technosignature persistence. The hazard of detectability loss is then λ_DL(t) = λ(t) × p_DL|war, where λ(t) is the nuclear‐exchange hazard estimated, and the corresponding survival function is S_DL(t) = exp(‐λ(t) × p_DL|war × t). The headline survival figures reported above implicitly set p_DL|war ≈ 1, treating nuclear exchange and detectability loss as effectively coincident. The nuclear‐winter and post‐catastrophe‐resilience literatures cited earlier suggest this is a defensible central case for severe exchanges under the high‐alert configurations to which our priors apply, but it rests on considerably thinner evidence than the hazard of exchange itself. Because median survival time scales as 1/p_DL|war for a fixed exchange hazard, setting p_DL|war = 0.5 rather than 1 would roughly double our median survival estimates; setting it well below 1 would weaken the paper's Fermi paradox implications correspondingly. We do not attempt to estimate p_DL|war formally in this article, since doing so credibly would require a second, largely independent hazard model linking conflict severity to long‐run technosignature persistence, for which Earth's single, non‐realized case provides essentially no calibrating data. We instead report results as conditional on p_DL|war ≈ 1 and identify the construction of an explicit p_DL|war model, potentially informed by the nuclear‐winter consequence literature (Robock et al. 2007, 2019; Toon et al. 2007, 2019), as a priority for future work rather than a feature of the present framework.

The Drake equation's longevity parameter *L*, that is, the duration civilizations remain detectable, critically determines expected observable civilization numbers. Traditional SETI analyses often assume *L* measured in thousands to millions of years. Our analysis suggests substantially shorter timescales under specific conditions.

Consider a civilization that becomes radio‐detectable at technological maturity and develops nuclear weapons contemporaneously (as occurred on Earth, separated by approximately 50 years). If it operates under high‐alert nuclear postures, our moderate scenario estimates median survival approximately 161 years from nuclear acquisition, with a 90% credible interval [11–2,181 years] (NUTS, N_CW  =  7). This figure is the median of the moderate Bayesian scenario distribution; it differs modestly from the deterministic plug‐in benchmark of 184 years for the reasons discussed there.

For Drake equation purposes, setting *L* = 161 years versus *L* = 1 million years reduces expected observable civilizations by a factor of approximately 6211. This dramatic reduction could partially resolve the Fermi paradox without requiring biological rarity.

However, this conclusion rests on three substantial assumptions: high‐alert nuclear configurations are common among technological civilizations, civilizations that survive the nuclear window do not subsequently achieve substantially longer lifespans, and other late‐stage filters do not dominate nuclear risks.

We therefore frame the SETI implication conditionally: if most civilizations develop under high‐vulnerability configurations and if these risks dominate subsequent survival, then brief technological lifespans could contribute to explaining SETI null results. If the nuclear window hypothesis holds under our specified scope conditions, several research directions follow, which we present as candidate lines of empirical inquiry rather than as firm, independently testable predictions, since each remains insufficiently developed to stand on its own. These directions include: biosignature‐technosignature divergence (atmospheric biosignatures might prove more common than technosignatures, supporting late‐stage filter localization; it might be testable with next‐generation telescopes), absence of galaxy‐scale engineering (very few civilizations should achieve Kardashev Type III capability, as this requires survival far exceeding nuclear window timescales), temporal clustering of signals (any detected technosignatures should show characteristics of civilizations in their brief technological windows), and archaeological signatures (atmospheric anomalies indicating past technological activity followed by collapse might outnumber active signals).

We emphasize these are conditional predictions contingent on assumptions about filter universality. Current SETI null results remain consistent with multiple explanations. The nuclear window provides one potential contributing factor rather than a complete resolution.

## 7 Conclusion

This article has developed a time‐varying hazard rate model to estimate civilizational survival under nuclear threat, using Earth's documented close calls as an illustrative case. Our key findings, properly caveated, are four. First, under plausible parameter ranges, civilizations operating under high‐alert nuclear postures face annual hazard rates on the order of 10^−^^3^–10^−^^2^, implying median survival times measured in decades to centuries. As illustrated in Figure 1, posterior distributions remain right‐skewed but consistently concentrated far above natural background extinction hazards. Even allowing for wide credible intervals, the order‐of‐magnitude separation persists. Second, temporal non‐stationarity matters critically. Post‐Cold War hazards appear substantially reduced, though limited observation time provides only weak constraints on current risk levels. Third, these findings are conditional on specific institutional configurations: geopolitical fragmentation, launch‐on‐warning postures, early‐generation early warning systems, and centralized command authority. Fourth, if such configurations prove common among technological civilizations, this could partially contribute to explaining the Fermi paradox by reducing Drake equation parameter L from millions of years to centuries.

For humanity, these findings underscore priorities in nuclear risk reduction: de‐alerting initiatives reducing launch‐on‐warning postures could substantially decrease hazard rates; early warning system redundancy and reliability improvements can reduce false alarm frequency; crisis communication channels and verification mechanisms may reduce escalation probability; nuclear proliferation to states with less robust command‐and‐control systems may increase systemic risk.

Current investment in existential risk reduction (Ord estimates less than 0.001% of GWP) appears dramatically insufficient relative to estimated hazard magnitudes. Even under deep uncertainty, the orders‐of‐magnitude difference between anthropogenic and natural risks justifies substantially increased resource allocation. These results suggest that anthropogenic risks may plausibly exceed natural background hazards by multiple orders of magnitude, a consideration relevant for risk‐prioritization debates.

This comparison is also relevant with recent work arguing that national and institutional risk assessments often omit or underweight global catastrophic and existential threats despite their potentially high salience under even conservative probability–impact assumptions (Boyd and Wilson 2023). Our contribution is narrower: rather than ranking all catastrophic risks, we estimate how one historically grounded anthropogenic hazard could shorten the detectable lifetime of technological civilization.

Beyond substantive findings, this article demonstrates methodological approaches for existential risk quantification under deep uncertainty: grounding estimates in formal stochastic processes rather than heuristic calculations, explicitly modeling temporal non‐stationarity through regime‐switching, using Bayesian scenario distribution structures to propagate parameter uncertainty through credible intervals, and framing conclusions conditionally with explicit scope limitations. These methods could extend to other existential risks where historical precedents are limited but structured analysis remains valuable.

Given these pros, the present analysis suffers of several limitations. First, the analysis rests on a sample size of n = 1 civilization observed during a single 80‐year period. This is not a robust empirical foundation for universal claims. We cannot know whether Earth's technological development pathway is typical or highly unusual, whether bipolar Cold War dynamics generalize to other civilizations, whether nuclear weapons represent a universal vulnerability or whether other technologies pose greater risks, or whether 80 years is sufficient observation time to characterize century‐scale or millennium‐scale risks.

Our methodology treats Earth as an illustrative case rather than a reliable statistical sample. The quantitative estimates should be interpreted as scenario explorations, demonstrating what might be true under certain conditions, rather than as empirical measurements of universal quantities.

Our analysis suffers from survivorship bias: we observe only the history of a civilization that (so far) has not destroyed itself. If nuclear windows typically last only decades and most civilizations fail quickly, we would not be here to observe such failures. The fact that humanity has survived 80 years might indicate that Earth's configuration is unusually safe rather than typical. This is a specific instance of the “anthropic shadow” identified by Ćirković et al. (2010): observation‐selection effects mean that catastrophes incompatible with the continued existence of observers are systematically underrepresented in any catalogue compiled by those observers, biasing close‐call counts and escalation‐probability priors alike toward optimism. As Ćirković (2012) argues, this kind of low‐probability, high‐consequence mechanism warrants serious analytical treatment even where theoretical confidence is limited, which is the posture we adopt throughout this article.

Furthermore, our close call catalogue may underestimate true danger: we observe only incidents where catastrophe was averted, often through individual heroism or fortunate chance. The counterfactual worlds where B‐59 launched its torpedo, or where Petrov reported the false alarm, or where Able Archer escalated; those worlds produced no observers to compile close call lists.

It is also important to highlight that nuclear self‐destruction is merely one candidate filter among many possibilities. These include abiogenesis (the origin of life itself might be extraordinarily rare; if Earth is the only planet in the galaxy with life, the Great Filter lies entirely in our past), eukaryotic transition (complex cells took approximately 2 billion years to evolve on Earth, suggesting this step is extremely difficult), intelligence evolution (tool‐using intelligence might be vanishingly rare even given multicellular life), other anthropogenic risks (engineered pandemics, misaligned artificial intelligence, nanotechnology accidents, or unforeseen technologies might pose risks comparable to or exceeding nuclear war), or economic filters (civilizations might systematically turn inward, devoting more and more time to virtual reality, computation, and or cultural pursuits, rather than pursuing space exploration, rendering them undetectable despite longevity).

Nonetheless, the nuclear window need not be the Great Filter to be important. Multiple filters might operate serially, each reducing survival probability. Even if nuclear risks constitute only one filter among several, they may still significantly shape L.

Finally, our Poisson process model with regime‐switching improves upon heuristic frequency calculations but retains significant simplifications. First, we made an independence assumption: we treat incidents as independent realizations. In reality, institutional learning, evolving command structures, and policy responses create temporal dependencies. Second, we assume constant p*_esc_* across incidents (homogeneous escalation probabilities). Acute political crises likely have higher p*_esc_* than technical false alarms. Third, we assumed sharp regime boundaries: our piecewise‐constant model assumes discontinuous hazard change at 1991. Gradual transition might better capture reality. Finally, with only 8 observed incidents (7 Cold War, 1 post‐1991), we cannot test this assumption against alternatives. More sophisticated models could address these limitations but would require substantially more data for reliable identification.

Two specific extensions follow directly from these simplifications and are worth naming rather than leaving subsumed under the general call for more sophisticated models. First, treating the eight close calls as independent Poisson arrivals likely understates the effective dependence among them, since several incidents are temporally and causally clustered: the Cuban Missile Crisis combined multiple near‐simultaneous near‐escalations, and the NORAD false alarm, the Petrov incident, and Able Archer 83 occurred within a four‐year span during a single period of heightened superpower tension. A self‐exciting (Hawkes) process, in which each incident temporarily raises the intensity of subsequent incidents, would more plausibly capture this clustering than the stationary or piecewise‐constant Poisson specifications used here, at the cost of additional parameters that our catalogue (N_CW  =  7, N_post  =  1) is poorly positioned to identify with any precision. Second, the assumption of a single, known regime boundary at 1991 is itself a simplification of the same kind: a continuous hazard‐decline specification, or a change‐point model with uncertain break location, would relax this assumption, but eight total incidents provide too little information to discriminate credibly among these alternatives via formal model comparison. We view both the Hawkes‐process and continuous‐decline extensions as natural directions for follow‐up work, appropriate once a larger or more granular incident catalogue becomes available, rather than as modifications we can responsibly implement on the present data without risking spurious precision.

The Fermi paradox asks: where is everybody? One possible answer, explored here under careful caveats, is that technological civilizations may commonly face a vulnerable period immediately following the technological threshold that makes them detectable. The same scientific and industrial capacities that enable interstellar communication also enable civilization‐scale destruction. If this coupling is common, the communicative phase of civilizations may be intrinsically short‐lived, sharply reducing the probability of temporal overlap between independent technological species. If institutional development lags technological capability, and if certain political‐technical configurations emerge commonly, then civilizational lifespans might be measured in decades to centuries rather than millions of years.

This hypothesis remains speculative: we cannot know from a sample of one whether Earth's trajectory is typical or exceptional. However, the documented historical record demonstrates that extinction‐level risks can arise quickly, operate frequently, and persist for extended periods. Each of Earth's eight nuclear close calls represented a moment where the thread of civilization hung by decisions of individuals, the reliability of early warning systems, and sometimes simply chance.

Whether humanity successfully navigates its nuclear window, and what this implies for intelligence elsewhere in the cosmos, remains one of the most consequential open questions facing our species. The profound silence we observe when we look to the stars may reflect not the rarity of intelligence but the fragility of technological civilizations, operating within narrow survival windows. Each year we survive provides weak evidence for navigability; each continued investment in risk reduction increases our probability of long‐term survival. The outcome of this uncertain experiment matters not only for understanding our place in the universe but for determining whether we will have a place in the universe at all.

## Disclosure on AI Policy

The author used ChatGPT 5.0 to improve readability and language.After using this tool, the author reviewed and edited the content as needed and takes full responsibility for the content of the article.

## Supporting information




**Supplementary File**: risa70339‐supp‐0001‐SuppMat.docx

## References

[risa70339-bib-0002] Barrett, A. M. , S. D. Baum , and K. R. Hostetler . 2013. “Analyzing and Reducing the Risks of Inadvertent Nuclear War Between the United States and Russia.” Science and Global Security 21, no. 2: 106–133.

[risa70339-bib-0003] Baum, S. D. , J. D. Haqq‐Misra , and S. D. Domagal‐Goldman . 2019. “Would Contact With Extraterrestrials Benefit or Harm Humanity?” Acta Astronautica 159: 323–330.

[risa70339-bib-0004] Beard, S. , T. Rowe , and J. Fox . 2020. “An Analysis and Evaluation of Methods Currently Used to Quantify the Likelihood of Existential Hazards.” Futures 115: 102469.

[risa70339-bib-0500] Baum, S. , R. de Neufville , and A. Barrett . March 8, 2018. A Model for the Probability of Nuclear War. Global Catastrophic Risk Institute Working Paper 18‐1, Available at SSRN. https://ssrn.com/abstract=3137081.

[risa70339-bib-0005] Blair, B. G. , H. A. Feiveson , and F. N. von Hippel . 1993. “Taking Nuclear Weapons off Hair‐Trigger Alert.” Scientific American 269, no. 5: 74–81.

[risa70339-bib-0006] Bostrom, N. 2002. “Existential Risks.” Journal of Evolution and Technology 9, no. 1: 1–31.

[risa70339-bib-0007] Bostrom, N. 2008. “Where Are They?” MIT Technology Review 111, no. 3: 72–77.

[risa70339-bib-0010] Boyd, M. , and N. Wilson . 2023. “Assumptions, Uncertainty, and Catastrophic/Existential Risk: National Risk Assessments Need Improved Methods and Stakeholder Engagement.” Risk Analysis 43: 2486–2502. 10.1111/risa.14123. 36907587

[risa70339-bib-0011] Carter, B. 2008. “Five‐ or Six‐Step Scenario for Evolution?” International Journal of Astrobiology 7, no. 2: 177–182.

[risa70339-bib-0012] Ćirković, M. M. 2012. “Small Theories and Large Risks—Is Risk Analysis Relevant for Epistemology?” Risk Analysis 32: 1994–2004. 10.1111/j.1539-6924.2012.01914.x. 23078410

[risa70339-bib-0013] Ćirković, M. M. , A. Sandberg , and N. Bostrom . 2010. “Anthropic Shadow: Observation Selection Effects and Human Extinction Risks.” Risk Analysis 30, no. 10: 1495–1506. 20626690 10.1111/j.1539-6924.2010.01460.x

[risa70339-bib-0015] Cox, D. R. 1972. “Regression Models and Life‐tables.” Journal of the Royal Statistical Society: Series B 34, no. 2: 187–202.

[risa70339-bib-0016] Denkenberger, D. , and J. M. Pearce . 2014. Feeding Everyone No Matter What. Academic Press.

[risa70339-bib-0502] Denkenberger, D. C. , and J. M. Pearce . 2015. “Feeding Everyone: Solving the Food Crisis in Event of Global Catastrophes That Kill Crops or Obscure the Sun.” Futures 72: 57–68. 10.1016/j.futures.2014.11.008.

[risa70339-bib-0017] Diamond, J. 2005. Collapse: How Societies Choose to Fail or Succeed. Viking Press.

[risa70339-bib-0018] Drake, F. D. 1961. “The Radio Search for Intelligent Extraterrestrial Life.” In Current Aspects of Exobiology, edited by G. Cameron , 323–345. Pergamon Press.

[risa70339-bib-0019] Fearon, J. D. 1995. “Rationalist Explanations for War.” International Organization 49, no. 3: 379–414.

[risa70339-bib-0021] Futter, A. 2015. The Politics of Nuclear Weapons. SAGE Publications.

[risa70339-bib-0024] Hanson, R. 1998. The Great Filter—Are we Almost Past it? http://mason.gmu.edu/~rhanson/greatfilter.html.

[risa70339-bib-0025] Haqq‐Misra, J. , R. K. Kopparapu , and E. Schwieterman . 2020. “Demarcating Biosignatures and Technosignatures.” Astrobiology 20, no. 4: 572–578. 32364797 10.1089/ast.2019.2154

[risa70339-bib-0026] Hellman, M. 2008. Risk Analysis of Nuclear Deterrence. The Bent of Tau Beta Pi, Spring, 14–22.

[risa70339-bib-0027] Jervis, R. 1979. “Deterrence Theory Revisited.” World Politics 31, no. 2: 289–324.

[risa70339-bib-0028] Jervis, R. 1989. The Meaning of the Nuclear Revolution. Cornell University Press.

[risa70339-bib-0501] Jiang, J. H. , R. Huang , and P. Das , et al. 2022. Avoiding the Great Filter: A Simulation of Important Factors for Human Survival [Preprint]. *arXiv* . 10.48550/arXiv.2209.02396.

[risa70339-bib-0030] Kalbfleisch, J. D. , and R. L. Prentice . 2002. The Statistical Analysis of Failure Time Data. 2nd ed. Wiley.

[risa70339-bib-0031] Kallenborn, Z. , and H. H. Willis . 2025. “Globally Critical Infrastructure: The Unique Risks and Challenges.” Risk Analysis 45, no. 12: 4804–4817. 10.1111/risa.70147. 41219065 PMC12747701

[risa70339-bib-0032] Kent, A. 2004. “A Critical Look at Risk Assessments for Global Catastrophes.” Risk Analysis 24, no. 1: 157–168. 15028008 10.1111/j.0272-4332.2004.00419.x

[risa70339-bib-0036] Lundgren, C. 2013. “What Are the Odds? Assessing the Probability of a Nuclear War.” The Nonproliferation Review 20, no. 2: 361–374. 10.1080/10736700.2013.799828.

[risa70339-bib-0037] McAnany, P. A. and Yoffee, N. eds. 2010. Questioning Collapse. Cambridge University Press.

[risa70339-bib-0038] Motesharrei, S. , J. Rivas , and E. Kalnay . 2014. “Human and Nature Dynamics (HANDY).” Ecological Economics 101: 90–102.

[risa70339-bib-0039] ÓhÉigeartaigh, S. O. , S. Beard , M. Rees , et al. 2020. “Overcoming the Challenges of Predicting Existential Risks.” Futures 115: 102469.

[risa70339-bib-0040] Ord, T. 2020. The Precipice: Existential Risk and the Future of Humanity. Bloomsbury Publishing.

[risa70339-bib-0041] Ord, T. , R. Hillerbrand , and A. Sandberg . 2010. “Probing the Improbable: Methodological Challenges for Risks With Low Probabilities and High Stakes.” Journal of Risk Research 13, no. 2: 191–205.

[risa70339-bib-0042] Piovani, D. , G. K. Nikolopoulos , and S. Bonovas . 2021. “Pitfalls and Perils of Survival Analysis Under Incorrect Assumptions: The Case of COVID‐19 Data.” Biomédica 41, no. 2: 21–28. 34669275 10.7705/biomedica.5987 PMC8582431

[risa70339-bib-0044] Powell, R. 1990. Nuclear Deterrence Theory. Cambridge University Press.

[risa70339-bib-0045] Rhodes, R. 2007. Arsenals of Folly: The Making of the Nuclear Arms Race. Knopf.

[risa70339-bib-0046] Robock, A. , L. Oman , and G. L. Stenchikov . 2007. “Nuclear Winter Revisited with a Modern Climate Model and Current Nuclear Arsenals: Still Catastrophic Consequences.” Journal of Geophysical Research: Atmospheres 112, no. D13: D13107. 10.1029/2006JD008235.

[risa70339-bib-0047] Robock, A. , O. B. Toon , and R. P. Turco . 2019. “Still Catastrophic Consequences.” Journal of Geophysical Research: Atmospheres 124, no. 15: 8522–8543.

[risa70339-bib-0049] Sagan, S. D. 1993. The Limits of Safety: Organizations, Accidents, and Nuclear Weapons. Princeton University Press.

[risa70339-bib-0052] Sandberg, A. , E. Drexler , and T. Ord . 2018. “Dissolving the Fermi Paradox.” Preprint, June 6, 10.48550/arXiv.1806.02404.

[risa70339-bib-0053] Schelling, T. C. 1960. The Strategy of Conflict. Harvard University Press.

[risa70339-bib-0054] Schelling, T. C. 1966. Arms and Influence. Yale University Press.

[risa70339-bib-0057] Tainter, J. A. 1988. The Collapse of Complex Societies. Cambridge University Press.

[risa70339-bib-0058] Toon, O. B. , A. Robock , R. P. Turco , C. Bardeen , L. Oman , and J. Robock . 2019. “Rapidly Expanding Nuclear Arsenals in Pakistan and India Portend Regional and Global Catastrophe.” Science Advances 5, no. 10: eaay5478. 31616796 10.1126/sciadv.aay5478 PMC6774726

[risa70339-bib-0059] Toon, O. B. , A. Robock , and R. P. Turco . 2007. “Environmental Consequences of Nuclear War.” Physics Today 60, no. 12: 37–42.

[risa70339-bib-0060] Wallace, M. D. , B. L. Crissey , and L. I. Sennott . 1986. “Accidental Nuclear War: A Risk Assessment.” Journal of Peace Research 23, no. 1: 9–27.

[risa70339-bib-0061] Webb, S. 2015. If the Universe Is Teeming With Aliens… Where Is Everybody? 2nd ed. Springer.

[risa70339-bib-0062] Wright, J. T. , B. Mullan , S. Sigurdsson , and M. S. Povich . 2014. “The Ĝ Infrared Search for Extraterrestrial Civilizations.” The Astrophysical Journal 792, no. 1: 26.

