# Supplementary material for: When Communication Enables Catastrophe: Nuclear Risk and the Short Lifetimes of Technological Civilizations: A Bayesian Scenario Analysis of Civilizational Survival and the Fermi Paradox
Source: Risk Anal. 2026 Aug 21;46(9):e70339. doi: 10.1111/risa.70339 (PMC13494566; doi:10.1111/risa.70339)
Supplement: Supplementary file 1 [file RISA-46-0-s001.docx]

# Appendix A – Catalogue of cases

This appendix documents the empirical foundation underlying the hazard modeling presented in the main text. It assembles the complete catalogue of historically documented nuclear close calls between 1950 and 1995 and provides extended analytical discussion of the escalation mechanisms most relevant for probabilistic risk estimation. The purpose is evidentiary rather than narrative: to ground prior specification, escalation-probability ranges, and regime-dependent hazard assumptions in the historical record while preserving analytical clarity in the main text.

The earliest episode considered arises during the Korean War, when Chinese intervention and the advance of United Nations forces toward the Yalu River led United States leadership to contemplate nuclear weapons use publicly and within formal military planning. Senior commanders advocated tactical atomic strikes against Chinese forces and bases in Manchuria, and planning documents envisioned dozens of detonations. Because the Soviet Union had already tested nuclear weapons and maintained alliance commitments with China, any such use carried the potential for escalation into superpower nuclear exchange. Despite this danger, deliberative political decision-making and the limited size of the Soviet arsenal suggest a moderate-to-low escalation probability.

A related but distinct confrontation occurred during the 1956 Suez Crisis. Anglo-French-Israeli military action against Egypt prompted explicit Soviet nuclear threats directed at Western powers. These warnings, preserved in diplomatic and archival records, were sufficiently credible to influence withdrawal decisions, even though they likely represented brinkmanship rather than immediate operational readiness. Escalation risk therefore appears low to moderate, illustrating how nuclear coercion can shape outcomes even without imminent launch preparations.

The Cuban Missile Crisis of October 1962 constitutes the most acute nuclear confrontation in recorded history and provides the clearest illustration of how multiple escalation pathways can converge. Reconnaissance evidence of Soviet missile deployment in Cuba created a compressed strategic decision environment in which invasion, air strikes, and blockade were all seriously considered. The most dangerous moment occurred on 27 October, when the Soviet submarine B-59, isolated under depth-charge signaling and unable to communicate with Moscow, prepared to launch a nuclear torpedo. Launch required the consent of three officers, and authorization was ultimately prevented only by the refusal of Vasily Arkhipov, whose dissent forced the submarine to surface and await orders. Subsequent historical reconstruction indicates that nuclear torpedo detonation would likely have triggered invasion, tactical nuclear use, and eventual strategic exchange, implying a high escalation probability during peak crisis conditions. Additional near-escalations—including a U-2 shootdown, accidental overflight into Soviet airspace, and severe communication delays—demonstrate that several independent failure channels operated simultaneously. The episode therefore reveals the importance of contingent individual judgment, hidden adversary capabilities, and clustered near-failures for hazard estimation.

Escalation risk reappeared during the 1973 Yom Kippur War, when regional conflict intersected with superpower confrontation and heightened alert levels. Evidence suggests possible Israeli nuclear readiness and a United States DEFCON 3 alert in response to Soviet intervention threats, producing compressed decision timelines and elevated miscalculation risk. Although archival opacity limits certainty, the overall escalation probability is plausibly moderate.

A qualitatively different pathway emerged in November 1979, when a NORAD training tape was mistakenly introduced into an operational warning system, generating the false appearance of a massive Soviet missile strike. Retaliatory forces were activated within minutes before cross-checking sensors prevented launch. This episode demonstrates that escalation risk can originate in technical malfunction rather than adversarial intent, with redundancy and verification acting as partial safeguards.

Technical vulnerability became even clearer in September 1983, when Soviet early-warning satellites falsely reported incoming United States missiles. Lieutenant Colonel Stanislav Petrov, serving as duty officer, judged the signal erroneous and declined to report an attack up the command chain, thereby violating formal protocol. Ground radar confirmation later proved him correct. The incident occurred amid extreme geopolitical tension, degraded communication, and launch-on-warning doctrine that compressed decision time to minutes. Scholarly interpretations of the counterfactual range from negligible to substantial escalation probability, but all agree that correct system behavior depended on discretionary human judgment rather than institutional robustness.

Only weeks later, the Able Archer 83 NATO exercise generated another escalation pathway rooted not in malfunction or deliberate aggression but in misperception. Soviet intelligence interpreted realistic exercise procedures as possible preparation for genuine nuclear attack and placed forces on heightened readiness, while NATO remained unaware of Soviet alarm. The resulting asymmetry in threat perception created a fragile environment in which unrelated incidents could have triggered escalation. Retrospective intelligence assessments concluded that relations may inadvertently have approached hair-trigger conditions. This case illustrates that crises can emerge without conscious recognition and that escalation risk may arise from informational mismatch alone.

The final documented close call occurred in January 1995, when a Norwegian scientific rocket was misidentified by Russian early-warning systems as a submarine-launched ballistic missile. Nuclear command procedures were activated, and national leadership had only minutes to determine whether retaliation was required before trajectory recalculation revealed the launch to be benign. Although escalation probability appears low to moderate, the episode demonstrates the persistence of misidentification risk even after the Cold War.

Table A1 synthesizes historically documented incidents forming the empirical basis of the hazard analysis. Evidence quality reflects availability of declassified documentation and scholarly reconstruction. Escalation-risk categories are qualitative and serve to motivate probabilistic prior ranges rather than provide direct frequency estimates.

Taken together, these incidents reveal several structural regularities relevant for probabilistic hazard modeling. All documented close calls cluster within the Cold War period, followed by an extended absence of comparable events, indicating regime-dependent rather than stationary risk. Escalation mechanisms are heterogeneous, spanning deliberate brinkmanship, technical malfunction, and misinterpretation, implying that vulnerability arises from interacting failure modes rather than a single dominant cause. Individual human decisions repeatedly prove decisive in preventing catastrophe, highlighting contingency within ostensibly rigid command systems. Finally, escalation risk is intensified by extreme compression of decision timelines—from minutes in technical false alarms to hours or days in political crises—far shorter than ordinary strategic deliberation. These regularities justify modeling nuclear danger as a time-varying, mechanism-diverse civilizational hazard shaped by institutional structure and temporal urgency rather than random fluctuation alone.

Table A2 formalizes the inferential bridge between qualitative historical evidence and quantitative Bayesian prior specification. The intent is not to derive parameter values directly from historical frequency, but to constrain plausible ranges consistent with documented escalation dynamics and institutional behavior. Such historically anchored yet uncertainty-forward priors are standard practice in risk analysis under deep uncertainty.

Table A1 – Incidents

| **Incident** | **Date** | **Trigger Type** | **Core Escalation Pathway** | **Decision Window** | **Evidence Quality** | **Qualitative Escalation Risk** |
| --- | --- | --- | --- | --- | --- | --- |
| Korean War nuclear threats | 1950–1951 | Political crisis | Consideration of U.S. nuclear strikes with potential Soviet retaliation | Days–months | High | Moderate–low |
| Suez Crisis | 1956 | Political brinkmanship | Explicit Soviet nuclear threats influencing Western withdrawal | Days | High | Low–moderate |
| Cuban Missile Crisis | Oct 1962 | Acute geopolitical confrontation | Nuclear torpedo launch nearly authorized; multiple simultaneous near-escalations | Minutes–days | Highest | High |
| Yom Kippur War | Oct 1973 | Regional war with superpower alert | Possible Israeli nuclear readiness and U.S.–Soviet confrontation | Hours–days | Medium–high | Moderate |
| NORAD false alarm | Nov 1979 | Technical system failure | Erroneous missile warning triggered rapid retaliatory preparations | Minutes | High | Low–moderate |
| Petrov incident | Sep 1983 | Technical false alarm | Protocol violation by duty officer prevented escalation under launch-on-warning posture | Minutes | High | Low to moderate (uncertain) |
| Able Archer 83 | Nov 1983 | Misinterpreted military exercise | Soviet alert triggered by perceived NATO first-strike preparation | Days | High | Low but elevated |
| Norwegian rocket incident | Jan 1995 | Misidentification of scientific launch | Russian nuclear command activated before trajectory reclassification | Minutes | High | Low–moderate |

Table A2 – Mapping Historical Incidents to Prior Parameter Justification

| **Prior Parameter** | **Empirical Motivation from Incidents** | **Direction of Constraint** | **Implication for Prior Choice** |
| --- | --- | --- | --- |
| Escalation probability (p*_esc_*) — upper bound | Cuban Missile Crisis shows multiple simultaneous near-escalations and contemporaneous leadership belief in substantial war probability | Raises plausible upper tail | Supports allowance for crisis-level escalation probabilities approaching order 10⁻¹ |
| Escalation probability (p*_esc_*) — lower bound | Technical false alarms (NORAD, Petrov) resolved through verification or individual judgment without launch | Prevents near-zero assumption | Justifies lower bound above zero, typically ≥10⁻³ |
| Heterogeneity of p*_esc_* | Distinct mechanisms: political confrontation, technical malfunction, and misperception (Able Archer) | Implies wide dispersion | Motivates log-scale or heavy-tailed prior rather than narrow distribution |
| Catalogue completeness (α) | Partial archival access, classification asymmetries, and definitional ambiguity of “close call” | Suggests systematic undercounting | Supports symmetric but weakly informative Beta prior centered below full observability |
| Regime hazard reduction (r = λ_post_ / λ_CW_) | Temporal clustering of most major incidents in the Cold War period, with a single incident meeting criteria in the post-1991 period (N_CW = 7, N_post = 1) | Indicates structural decline in risk | Justifies prior mass on r < 1 while allowing incomplete risk elimination |
| Independence assumptions | Repeated reliance on individual discretionary decisions (Arkhipov, Petrov) and institutional learning after crises | Suggests partial dependence | Encourages conservative uncertainty rather than precise frequency inference |
| Decision-time compression | Minute-scale response windows in technical alarms versus longer political crises | Links mechanism to escalation likelihood | Supports modeling escalation probability as mechanism-dependent and highly uncertain |

**Appendix B - Posterior Sampling Diagnostics**

Posterior sampling was performed using the No-U-Turn Sampler (NUTS) implemented in PyMC 6.0. Four independent Markov chains were run for each prior scenario, each consisting of 2,000 warm-up iterations followed by 3,500 retained draws, with a target acceptance probability of 0.95.

Convergence was assessed using split R, bulk and tail effective sample sizes, divergent-transition counts, trace plots, and rank histograms. All monitored quantities achieved R=1.000. Bulk ESS ranged from 5,967 to 12,613 and tail ESS from 3,299 to 8,968, with no divergent transitions. These diagnostics provide no evidence of convergence or sampling problems.

Figures S1–S3 present trace plots and marginal posterior densities for the three primary model parameters. The four chains overlap closely, show no visible trends or chain-specific behavior, and exhibit stable stationary sampling throughout.

Figures S4–S6 present rank histograms for the same parameters. The approximately uniform distributions indicate that all chains explored the same posterior distribution and provide no evidence of poor mixing or convergence problems.

Together, these diagnostics indicate satisfactory posterior exploration and reliable estimation of the posterior summaries reported in the main text.

### Figure S1 - Trace plots and marginal posterior densities for the Optimistic scenario.

Four independent NUTS chains are shown for the three sampled parameters. The absence of visible trends or chain separation indicates satisfactory mixing.

### Figure S2 - Trace plots and marginal posterior densities for the Moderate scenario.

Four independent NUTS chains are shown for the three sampled parameters. The absence of visible trends or chain separation indicates satisfactory mixing.

### Figure S3 - Trace plots and marginal posterior densities for the Pessimistic scenario.

Four independent NUTS chains are shown for the three sampled parameters. The absence of visible trends or chain separation indicates satisfactory mixing.

*Figure S4 - Rank histograms for the Optimistic scenario.*

Approximately uniform rank distributions across all four chains indicate satisfactory convergence and mixing.

*Figure S5 - Rank histograms for the Moderate scenario.*

Approximately uniform rank distributions across all four chains indicate satisfactory convergence and mixing.

*Figure S6 - Rank histograms for the Pessimistic scenario.*

Approximately uniform rank distributions across all four chains indicate satisfactory convergence and mixing.
